# Prediction model of interaction anxiousness based on Weibo data

**DOI:** 10.3389/fpubh.2022.1045605

**Published:** 2022-11-08

**Authors:** Yilin Wang, Nan Zhao

**Affiliations:** ^1^CAS Key Laboratory of Behavioral Science, Institute of Psychology, Chinese Academy of Sciences, Beijing, China; ^2^Department of Psychology, University of Chinese Academy of Sciences, Beijing, China

**Keywords:** interaction anxiousness, mental health, machine learning, non-invasive identification, adolescent

## Abstract

Adolescents who face social distress in real life are often accompanied by interaction anxiousness. To avoid direct social activities, they prefer to indulge in social networks to satisfy their psychological needs for interpersonal communication. Sina Weibo, China's leading social media platform, has a markedly young user base. It provides a rich sample of adolescents with interaction anxiousness and conditions for real-time monitoring. In this study, various word categories, such as perception of spatial distance and positional relationships, morality, and emotion, showed a significant relationship with interaction anxiousness. Furthermore, prediction models were established based on the original Weibo data of 839 active Sina Weibo users through a variety of machine learning algorithms to predict the scores of users' interaction anxiousness. The results showed that the performance of the prediction model established by the fully connected neural network was the best, and both criterion validity and split-half reliability were good (*r*_criterionvalidity_ = 0.30, *r*_split − halfreliability_ = 0.76). This study confirms the validity of the prediction model of interaction anxiousness based on social media behavior data, provides a feasible solution to examine adolescents' interaction anxiousness, and provides a scientific basis for more targeted mental health interventions.

## Introduction

Interpersonal communication is a unique and necessary daily psychological need of human beings as social animals (1, 2). From the perspective of real-life experience, good interpersonal communication is of great significance in shaping mental health (3–6). As the cost of socializing gradually decreases, the opportunities for interpersonal communication are increasing day by day. Nevertheless, many individuals in such situations suffer from interaction anxiousness, which damages their social ability and threatens their mental health (7).

As a common form of anxiety, interaction anxiousness refers to the emotional reaction and avoidance behavior of strong anxiety, excessive fear, nervousness, and even fear of one or more interpersonal situations. It is characterized by an intense fear of communicating face-to-face with others and negative evaluations from others in social situations (8–10). Interaction anxiousness has a high incidence and long duration and is more common in adolescence (11–13). Additionally, cross-cultural research shows that young people in Asia are more prone to interaction anxiousness (9). Interaction anxiousness will not only affect an adolescent's psychosocial adaption to real society but also induce a series of internalizing and externalizing problems (14). More seriously, it might induce social anxiety disorder, which adversely affects many aspects of adolescents' actual lives and even their mental health and achievements in adulthood (15, 16). Therefore, real-time and long-term monitoring of an adolescent's interaction anxiousness is conducive to maintaining the harmony and stability of his/her interpersonal communication status, the effectiveness and pertinence of psychological intervention at a micro level, and of significant importance for adjusting policies related to caring for adolescents promptly at a macro level.

At present, the traditional methods of measuring interaction anxiousness mostly revolve around self-reported inventories. There is a certain lag in real-time and long-term monitoring, and it is difficult to obtain timely responses from participants (17, 18). In addition, adolescents with interaction anxiousness are mostly in a youth rebellion period, and they tend to have a low degree of cooperation with strong resistance. The traditional method relies on active participation, so it is difficult to carry out the evaluation of interaction anxiousness and even brings extra burdens for participants (19).

As a leading social media platform in China, Sina Weibo provides a large sample of adolescents (20). With the rise of interdisciplinary research, many studies have confirmed the feasibility, accuracy, and reliability of identifying individual psychological characteristics based on social media behavior data, such as emotions and attitudes (21–23), personality (24), and mental health status (25). Online social media, a new platform to offer online interaction and communication, reduces the level of self-exposure and provides more communication opportunities for people with interaction anxiousness who are afraid of face-to-face communication with others (26). Individuals who face social distress in real life will rely more on online social media to meet their psychological needs for interpersonal communication (27, 28). In addition, individuals with interaction anxiousness tend to show more authentic selves online compared to offline interpersonal interactions (29, 30).

Consequently, if we could make use of the prediction model based on social media behavior data, it would be much more timely and convenient to assess the interaction anxiousness. Establishing a machine learning prediction model could be used as a viable method to identify individuals' psychological characteristics, which is worthy of study. Moreover, existing model evaluation methods, such as accuracy or mean square error, fail to meet the requirements of evaluating psychological index measurement tools (31). Therefore, we could try to apply the assessment method of psychological scales, such as reliability and validity, to machine learning models. As researchers often use the correlation between the scores of a certain scale with those of other scales to evaluate the quality of a scale (32, 33), we used the correlation between the predicted scores from models and actual scores from the scale to calculate validity, and we used the correlation between predicted scores from models based on the two halves of the Weibo posts to test split-half reliability.

In this study, in order to realize the long-term and timely detection of adolescents' interaction anxiousness, we made use of machine learning methods, using the data of Sina Weibo active users, to train mental prediction models so as to realize the automatic identification of interaction anxiousness. The aim of this study is to improve the implementation scale, time span, and testing efficiency of traditional methods and to provide a basis for non-intrusive automatic identification of adolescents and even the general public's social interaction and intervention projects.

## Methods

### Participants and data collection

This study used an online experiment platform to collect data on Sina Weibo users (25). In September 2012, a total of 1,846 Sina Weibo users who volunteered to participate in the experiment were recruited for this study. Since the Interaction Anxiousness Scale asks about the feelings of the subjects in social interaction consistently and does not limit the time range, this psychological characteristic is of trait partially (11). Therefore, in this study, we downloaded those users' original Weibo data within 1 year before the experiment was launched, so as to obtain relatively stable Weibo behaviors. In order to ensure the quality of the users' data, this study first filtered out meaningless posts, such as advertisement-related, simple forwarding, etc., and then removed non-text information such as emojis from the text content of posts.

Second, to effectively eliminate the data of inactive users, we selected active users who met the following criteria: (a) Published at least 500 original Weibo posts before the experiment; (b) The total word count of original Weibo posts before the experiment had been at least 5,000 words; and (c) The recent Weibo post must be published within 3 months before the experiment was launched.

Moreover, to ensure the quality of the annotation of the psychological characteristics of the users, this study also excluded the data of the users who filled in questionnaires in <30 s.

After these selection processes, the final sample contained 839 qualified users.

This study received ethical approval from the Institutional Review Board of the Institute of Psychology, Chinese Academy of Sciences with the ethics approval number H16003.

### Measurement and procedures

#### Interaction anxiousness

In this study, the Interaction Anxiousness Scale (IAS) was used to measure the tendency of individuals to experience subjective interaction anxiety. This scale shows a high correlation between anxiety and anxiety in real interpersonal situations and has good reliability and validity (34). Compared with low scorers, high scorers are more anxious and less confident before and during social interpersonal interactions, more concerned with how they are perceived in interactions and are also likely to feel more inhibited during conversations. The IAS consists of 15 items in total, which are about a series of descriptions related to subjective anxiety feelings in social interpersonal communication. For each item, respondents are asked to indicate the “degree to which the statement is characteristic or true of you” on a five-point Likert-Type scale (1 = “not at all,” 5 = “extremely characteristic”). The Cronbach's alpha of the IAS is beyond 0.87. The score range is 15–75 points; the higher the score, the more severe the interaction anxiousness of the subject.

#### Linguistic expression feature

We employed the Text Mind system developed by the Computational Cyber Psychology Laboratory at the Institute of Psychology, Chinese Academy of Sciences, to extract content features, including Chinese word segmentation tools, and psychoanalytic dictionaries (35). After the Chinese word segmentation tool was used to divide users' original Weibo post content into independent words/phrases with linguistic annotations (verbs, nouns, etc.) (36, 37), Simplified Chinese Language Inquiry and Word Count dictionary (SCLIWC) (38), Moral Motivation Dictionary (39), Moral Foundations Dictionary (40), Individualism/Collectivism Dictionary (41), Suicide Dictionary (42), and Weibo Five Basic Mood Lexicon (43) were used for linguistic expression feature extraction. Finally, a total of 120 linguistic expression features were obtained.

### Statistical analysis

#### Correlation analysis

To investigate the relationship between social media behaviors and psychological characteristics, we examined the Pearson correlation coefficient between each linguistic expression feature and interaction anxiousness.

#### Data modeling

This study adopted the machine learning methods of Multiple Linear Regression (MLR), Fully Connected Neural Network (FCNN), NuSVR in Support Vector Regression (SVR), and Extra Trees Regressor to establish prediction models of interaction anxiousness based on network data analysis respectively, and 10-fold cross-validation was used in all these prediction models to adjust model parameters. The evaluation indicators of the modeling effect included criterion validity and split-half reliability.

##### Criterion validity

The actual scores of the IAS were used as the criterion, and the Pearson correlation coefficient between the predicted scores from the model and the actual scores from the IAS was calculated as an indicator of criterion validity.

##### Split-half reliability

We sorted all qualified users according to the number of Weibo posts they published within 1 year before the experiment was launched, from high to low, and took the top 200 users' data as the test set and the rest as the training set and saved the model. Then, we sorted Weibo posts published by each user in the test set according to the order of release time and divided them into two parts as two split halves, the odd group and even group, based on the parity corresponding to the release time sorting. The Pearson correlation coefficient between the predicted scores of the two split-half data sets through the previously saved model was calculated as an indicator of split-half reliability.

## Results

### Demographic information

Among the 839 users mentioned above, 37.54% were men, and the average age was 23.08 ± 5.73 years. The educational level of the users (including those in progress) was mainly undergraduate (54.59%) and junior college (21.69%). The users were mainly college students (50.54%) and staff (20.62%). The per capita monthly household income was concentrated in the range of 2,000–4,000 yuan (37.66%) and 4,000–6,000 yuan (18.00%). Scores of interaction anxiousness ranged from 20 to 72, with an average score of 42.98 ± 9.83.

Table 1 features the demographic profiles of these participants.

**Table 1:** Demographic characteristics of participants.

	**Participants**
	***n* (%)**
**Educational level**	
Junior high	25 (2.98)
Senior high	96 (11.44)
Technical secondary school	42 (5.01)
Junior college	182 (21.69)
Undergraduate	458 (54.59)
Master	33 (3.93)
Doctor	3 (0.36)
**Occupation**	
Worker	13 (1.55)
Civil servant	13 (1.55)
Soldier	1 (0.12)
Researcher/teacher	33 (3.93)
Media employee	19 (2.26)
Farmer	3 (0.36)
Unemployed	26 (3.10)
Student	424 (50.54)
Doctor/nurse	16 (1.91)
Clerk	173 (20.62)
Freelancers	38 (4.53)
Self-employed	15 (1.79)
Others	65 (7.75)
**Per capita monthly household income**	
<2,000	131 (15.61)
2,000–4,000	316 (37.66)
4,000–6,000	151 (18)
6,000–8,000	108 (12.87)
8,000–10,000	54 (6.44)
>10,000	79 (9.42)
**Total**	839 (100.00)

### Relationship between linguistic expression features and interaction anxiousness

The results showed that 45 linguistic expression features had significant relationships with interaction anxiousness, including 31 features in SCLIWC (space and relative), two features in Moral Motivation Dictionary (agency and communion), seven features in Moral Foundations Dictionary (FairnessVirtue and MoralityGeneral), four features in Suicide Dictionary (B and C7), and one feature in Weibo Five Basic Mood Lexicon (happiness; as shown in Table 2).

**Table 2:** Pearson correlation coefficients between important behavioral features and interaction anxiousness.

**Linguistic expression feature**	**Explanation and example**	**Interaction anxiousness**	**Meaning of colors**
**LIWC**		
Linguistic processes		
Funct	Total function words (maybe, those, and these)	−0.074^*^	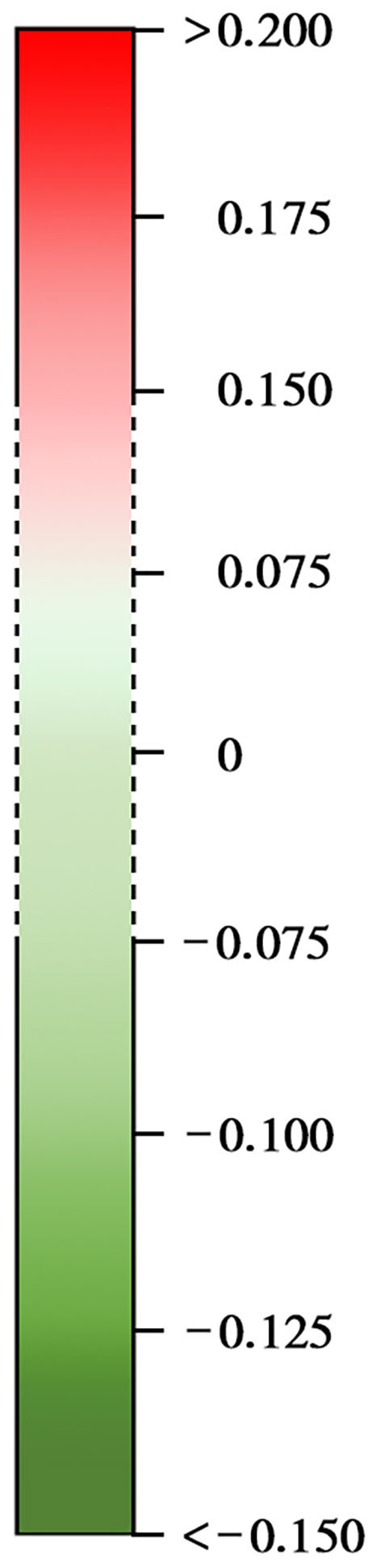
iPron	Impersonal pronouns (it, it's, and those)	−0.088^*^	
Verb	Common verbs (walk, went, and see)	−0.070^*^	
AuxVerb	Auxiliary verbs (am, will, and have)	−0.072^*^	
Preps	Prepositions (to, with, and above)	−0.147^***^	
Conj	Conjunctions (and, but, and whereas)	−0.104^**^	
Negate	Negations (no, not, and never)	−0.118^***^	
Quant	Quantifiers (few, many, and much)	−0.128^***^	
PrepEnd	Postposition (among, above, and till)	−0.080^*^	
MultiFun	Multifunction (of, be, and right)	−0.113^**^	
PastM	Signal words for past tense (yesterday and ago)	−0.070^*^	
PresentM	Signal words for present tense (often and usually)	−0.085^*^	
Social processes			
Humans	Humans (adult, baby, and boy)	−0.115^***^	
CogMech	Humans (adult, baby, and boy)	−0.099^**^	
Insight	Insight (think, know, and consider)	−0.137^***^	
Cause	Causation (because, effect, and hence)	−0.132^***^	
Tentat	Tentative (maybe, perhaps, and guess)	−0.090^**^	
Certain	Certainty (always and never)	−0.084^*^	
Inhibition	Inhibition (block, constrain, and stop)	−0.098^**^	
Exclusive	Exclusive (but, without, and exclude)	−0.100^**^	
Health	Health (clinic, flu, and pill)	−0.100^**^	
Sexual	Sexual (horny, love, and incest)	0.096^**^	
Relative	Relativity (area, bend, exit, and stop)	−0.098^**^	
Space	Space (down, in, and thin)	−0.117^***^	
Personal concern			
Work	Work (job, majors, and xerox)	−0.068^*^	
Achieve	Achievement (earn, hero, and win)	−0.070^*^	
Leisure	Leisure (cook, chat, and movie)	0.169^***^	
Religion	Religion (altar, church, and mosque)	−0.068^*^	
tPast	Past (recollect, recall, and memory)	−0.075^*^	
Spoken categories			
Filler	Fillers (blah, I mean, and you know)	−0.077^*^	
OtherP	Other punctuation	0.148^***^	
**Moral motivation**			
Agency	Agency (self, dominance, and achievement)	−0.099^**^	
Communion	Communion (benevolence, preservation, and hospitable)	−0.125^***^	
**Moral foundations**			
FairnessVirtue	Fairness (fairness, justice, and equality)	−0.128^***^	
FairnessVice	Deceit (deceive, immoral, and illegal)	−0.075^*^	
IngroupVirtue	Loyalty (homeland, group, and family)	−0.072^*^	
AuthorityVirtue	Authority (obedience, loyalty, and duty)	−0.093^**^	
AuthorityVice	Rebellion (revolt, provocation, and transgression)	−0.114^***^	
PurityVice	Dirty (disgust, corruption, and contagion)	−0.107^**^	
MoralityGeneral	Basic morality (evil, code, and integrity)	−0.140^***^	
**Suicide**			
B	Self-harm (self-immolation, self-mutilation, and self-injury)	−0.126^***^	
C3	Despair (wrong, ultimate, and dark)	−0.068^*^	
C6	Personality traits (impulsive, humble, and cowardly)	−0.083^*^	
C7	Stress (social, realistic, and bleak)	−0.144^***^	
**Weibo five basic mood**			
Happiness	Happiness (loving, overjoyed, and playful)	0.76^*^	

*** p < 0.001.

** p < 0.01.

* p < 0.05.

### Criterion validity

There were two prediction models established by MLR. In the first one, the prediction model was based on the 45 linguistic expression features mentioned above, which had a significant correlation with interaction anxiousness. In the second one, the largest feature in the whole feature set with a variance inflation factor (VIF) of over 10 was removed each time to avoid multicollinearity, and after 102 linguistic expression features with a VIF of <10 were retained, the prediction model of interaction anxiousness based on these retained features was established.

As for FCNN, after the tuning process of model parameters, this study set the learning rate of FCNN to 0.999, the dropout ratio to 0.3, the batch size to 64, and the training to 300 epochs. As for NuSVR and Extra Trees Regressor, the method of Random Forest was uniformly used for feature screening, and 46 features that had a greater impact on interaction anxiousness were reserved for further modeling. A 10-fold cross-validation was used in all these prediction models to adjust model parameters.

The results showed that for the same modeling target, the effects of prediction models based on different algorithms varied according to different correlation coefficients. Among them, the correlation coefficient between the predicted scores from the FCNN model and the actual IAS scores was the largest (*r* = 0.300), indicating that the FCNN prediction model had the highest criterion validity (as shown in Table 3).

**Table 3:** Criterion validity of interaction anxiousness model.

**Psychological characteristic**	**MLR_1_**	**MLR_2_**	**FCNN**	**NuSVR**	**Extra trees regressor**
Interaction anxiousness	0.122	0.146	0.300	0.215	0.201

MLR_1_ was based on features significantly related to interaction anxiousness, MLR_2_ was based on features whose VIF < 10. Correlation coefficients of prediction models were average results of 10-fold cross-validation.

### Split-half reliability

In view of the best performance of the interaction anxiousness prediction model established by the machine learning algorithm of FCNN, this study further calculated the split-half reliability of the FCNN model. We used the top 200 users with the number of Weibo posts published within 1 year before the experiment was launched from high to low as the test set, and we divided each user's Weibo posts into two parts according to the parity of their chronological order of his/her Weibo posts. After we established the FCNN model based on the rest of the users as the training set and saved it, we applied this FCNN model to the two split-half data sets of the test set to calculate the split-half reliability.

The Pearson correlation coefficient between the predicted scores of the two split-half data was calculated, which was used as the split-half reliability in the interaction anxiousness prediction model. The result showed that there was a significant correlation between the predicted scores of the two split-half data (*r* = 0.755, *p* < 0.001), indicating that the established prediction model of interaction anxiousness had high split-half reliability.

## Discussion

This study downloaded the original Sina Weibo data of active users, extracted linguistic expression features from it as the input, and collected users' corresponding IAS scores through an online survey as the output. We first explored the relationship between each linguistic expression feature and interaction anxiousness. Then we built prediction models of interaction anxiousness based on network data analysis. The results indicated that the criterion validity and split-half reliability of the FCNN model were good, demonstrating the model's accuracy and stability.

Through the investigation of the features that had a strong relationship with interaction anxiousness, we found several linguistic expression characteristics of individuals with high interaction anxiousness in social media, including grammatical habits, personal concerns, and so on. There were some characteristic patterns that might be able to bring meaningful inspiration to our understanding of interaction anxiousness:

### Perception of spatial distance and positional relationship

Individuals with higher interaction anxiousness mentioned less spatial distance (down, in, etc.) and fewer positional relationships (with, among, etc.). As individuals in social structures, we relate to others spatially and interpersonally (44). Maintaining an appropriate distance from others is important for effective communication and good relationships. Fewer related expressions may indicate some deficits in the perception of interpersonal distance in individuals with high interaction anxiousness. Previous research revealed that individuals with a social anxiety disorder might have a wrong perception of the distance required for social interaction; hence, they could not correctly grasp and skillfully use the concept of location information (45).

### Morality

Individuals with higher levels of interaction anxiousness were less likely to express morally relevant language. This highly relevant relationship existed not only in the positive dimension (fairness, communion, etc.), but also in the negative dimension (rebellion, dirty, etc.). These results are consistent with previous studies: When faced with interpersonal problems, individuals who internalize the moral concept of right and wrong can adopt more positive coping strategies and fewer negative reactions such as avoidance (46, 47). This finding suggests that strengthening moral concepts in thinking and expression may help to better cope with interpersonal situations and reduce interaction anxiousness.

### Emotion

Individuals with higher interaction anxiousness would express fewer negative emotions (stress, etc.) while expressing more positive emotions (happiness, etc.). This is in contrast to previous research: Individuals with interaction anxiousness can fall into a strong fear of how others perceive them (especially fear of embarrassment, criticism, or rejection). Then, they will have no sense of security, treat others unkindly and assume that peers automatically reject them, along with a negative emotional reaction (48). In this anxiety state, the positive subjective experience and hedonic activities of individuals in the real world will be reduced, and the expression of positive emotions will be inhibited (49, 50). The reason we infer the diametrically opposite findings is the different environments. Individuals who are deeply troubled by interaction anxiousness tend to ignore positive emotions and vent more negative emotions in real life, and this perception habit will further aggravate their interaction anxiousness (51). Individuals suffering from interaction anxiousness in real life tend to resort to the Internet for their needs for interpersonal communication and self-expression, and adjust, regulate, and change themselves to satisfy their needs. In contrast to real life, in an anonymous social network environment, individuals no longer fear being rejected by others, no longer worry about causing embarrassment and other negative reactions (such as laughing when no one finds it funny), and social avoidance behaviors are reduced. These allow individuals to have more cognitive resources to understand, accept, and express their true selves and focus on regulating their emotional experiences. At this time, they will have more positive experiences and fewer negative experiences (49). Therefore, individuals with more severe interaction anxiousness tend to use online social media to meet their communication needs and show their true selves, so that they can rebound from painful experiences and return to a hedonistic lifestyle (52).

Although there were several linguistic expression features related to interaction anxiousness, the Pearson correlation coefficients of them were relatively low, indicating that it was not feasible to predict interaction anxiousness through a single social media behavior. Therefore, we took the linguistic expression features as input and adopted machine learning methods, including linear and non-linear algorithms, to build prediction models to identify the psychological characteristics.

The criterion validity of this study showed that, compared with other machine learning algorithms, the FCNN model performed the best, the correlation coefficient between the predicted and the actual scores achieved a moderate level (53). It could be inferred that these linguistic expression features, in fact, had some non-linear relationship with interaction anxiousness. The split-half reliability showed that the predicted score of the FCNN model on different Weibo posts of the same user was highly correlated, indicating that the prediction model had satisfactory accuracy and stability. In general, the modeling effect of the prediction model of interaction anxiousness in this study is good, which provides a strong basis for the feasibility of the computational method of psychological characteristics based on network data analysis. This is also consistent with the previous study: Through the prediction model, records of users' linguistic expression features on online social media platforms can be used to identify their psychological characteristics (24, 54).

This study used the spontaneous Weibo data of online social media users in a non-intrusive situation to understand users' real interaction anxiousness state immediately, automatically, and continuously. This method of monitoring adolescent interaction anxiousness through social media behavior data is highly ecologically valid and brings new possibilities for controlling the status quo of social interpersonal communication. Moreover, since Sina Weibo not only records everyday real behaviors of users but also accumulates their long-term historical data, it could also be applied to tracking research of psychological characteristics, such as interaction anxiousness. The psychological modeling method adopted in this study could be traced back to a large time span, and research could be carried out at any recorded time point, which facilitates longitudinal observation (25, 55, 56).

Individuals who suffer from interaction anxiousness in real life are often more willing to use online social media to obtain some kind of psychological satisfaction (27–29), and they are also more likely to be online than offline to reveal their true inner selves (30). Online social media such as Sina Weibo, as the main way for adolescents to communicate with each other, provides an opportunity for information sharing and real-time interaction. The massive online data from social media not only provides a rich sample of adolescents with interaction anxiousness, but also roughly covers different regions or even different nations, which provides a reference for the development of highly representative and large-scale research (57).

As computer technology gathers momentum, the Internet has become an indispensable part of human life. In this study, a psychological characteristic prediction model based on social media behavior data provides a practical reference and in-depth research ideas for the traditional method of psychological characteristic identification. Through real-time and long-term monitoring of people's mental health, it is possible to quickly, timely, and low-cost grasp the psychosocial changes. This would contributive to many possible actions promoting the public's wellbeing, such as developing a more effective mental health service and making more relevant policies.

## Limitations

Although this study proves that it is feasible to build a prediction model of interaction anxiousness based on massive network data, there are still some deficiencies. First, in order to guarantee adequate text information and the quality of psychological scale completion, we had to eliminate some Sina Weibo users after the recruitment according to the criteria in the data filtering mentioned above. There is a limitation that we may not be able to represent the totality of active Sina Weibo users perfectly. Second, since the potential analysis objects of big data in artificial intelligence can only correspond to some groups (online social media users), there was a shortage of control over the overall psychological states of adolescents, and traditional methods still needed to be used to investigate the remaining population groups (non-network social media users) that have not yet been covered. Third, due to the data-driven method, the prediction model was difficult to systematically understand the deep-seated causes of changes in mental health. Therefore, it could be necessary to rely on the methodological advantages of traditional survey methods, such as interviews, to understand the underlying causes and specific manifestations of interaction anxiousness. Additionally, although the relationship between linguistic expression features and psychological characteristics is relatively stable over time, we cannot entirely exclude the possibility that the language expression style of users might have changed to some extent since we collected our dataset. In future studies, obtaining the latest data from today's Weibo users could be helpful.

## Conclusion

The purpose of this study was to explore the characteristic behavior patterns of individuals with a high level of interaction anxiousness, and determine whether an individual's psychological characteristic, interaction anxiousness, could be calculated in real-time through machine learning modeling. Based on the data of Sina Weibo, it was found that the criterion validity and split-half reliability of the machine learning model established by FCNN were good. That is, a psychological prediction model could be established by analyzing network data so as to realize the real-time calculation of the psychological characteristics among adolescents. This study provides a feasible method for assessing psychosocial changes in time to prevent the worsening of psychological problems.

## Data availability statement

The raw data cannot be made public. If necessary, we can provide feature data. Requests to access the datasets should be directed to NZ, zhaonan@psych.ac.cn.

## Ethics statement

The studies involving human participants were reviewed and approved by the Institutional Review Board of the Institute of Psychology, Chinese Academy of Sciences (H16003). Written informed consent to participate in this study was provided by the participants' legal guardian/next of kin. Written informed consent was obtained from the individual(s) and minor(s)' legal guardian/next of kin, for the publication of any potentially identifiable images or data included in this article.

## Author contributions

NZ conceived and planned the article. YW and NZ carried out the study and drafted the manuscript. YW developed the tools needed for the experiment, executed the whole experiment process, performed all of the statistical analyses, and wrote the manuscript with input from all authors. Both authors contributed to the article and approved the submitted version.

## Funding

This study was financially supported by the Strategic Priority Research Program of Chinese Academy of Sciences (No. XDC02060300), the Scientific Foundation of Institute of Psychology, Chinese Academy of Sciences (No. E2CX4735YZ), and Youth Innovation Promotion Association CAS.

## Conflict of interest

The authors declare that the research was conducted in the absence of any commercial or financial relationships that could be construed as a potential conflict of interest.

## Publisher's note

All claims expressed in this article are solely those of the authors and do not necessarily represent those of their affiliated organizations, or those of the publisher, the editors and the reviewers. Any product that may be evaluated in this article, or claim that may be made by its manufacturer, is not guaranteed or endorsed by the publisher.

## References

[1] World Health Organization. Preamble to the Constitution of the World Health Organization as Adopted by the International Health Conference, New York, 19 June- 22 July, 1946; Signed on 22 July 1946 by the Representatives of 61 States (Official Records of the World Health Organization, No. 2, p. 100) and Entered into Force on 7 April 1948. Geneva: WHO (1948).

[2] Cao H Cao P Wang P. A correlation study on coping styles and social anxiety in college students. J UESTC. (2009) 11:91–4.

[3] Liu M. Research on college students' relationship between interpersonal communication and mental health. J Hubei Adult Educ Inst. (2022)28:45–50. 35886470

[4] Qin L. Discussion on the optimization of body-mind coordination and the interpersonal relationships of college students. Heilongjiang Res High Educ. (2016) 11:129–31.

[5] Segrin C McNelis M Swiatkowski P. Social skills, social support, and psychological distress: a test of the social skills deficit vulnerability model. Hum Commun Res. (2015) 42:122–37. 10.1111/hcre.12070

[6] Solomon P. Peer support/peer provided services underlying processes, benefits, and critical ingredients. Psychiatr Rehabil J. (2004) 27:392–401. 10.2975/27.2004.392.401 15222150

[7] Li D Chen X Duan T Yu Z Yang Z. Interaction anxiousness and associated factors among college students. Chin J School Health. (2013) 34:835–7.

[8] American Psychiatric Association. Diagnostic and Statistical Manual of Mental Disorders. 5th ed. Arlington, VA: American Psychiatric Publishing (2013). 10.1176/appi.books.9780890425596

[9] Guo X. Study on cause of social anxiety. Explor Psychol. (2000) 20:55–8.

[10] Morrison AS Heimberg RG. Social anxiety and social anxiety disorder. Annu Rev Clin Psychol. (2013) 9:249–74. 10.1146/annurev-clinpsy-050212-185631 23537485

[11] Leary MR Kowalski RM. Social Anxiety. New York, NY: The Guilford Press (1995).

[12] Li W Liu L. Review of research on social-intercourse anxiety. Theory Practice Educ. (2007) 27:37–9. 10.1016/S0300-7073(07)70744-2

[13] Schmidt MH Blanz B. Anxiety syndromes in childhood and adolescence. Acta Paedopsychiatr. (1989) 52:42–9. 2697131

[14] Schutters SIJ Dominguez MDG Knappe S Lieb R van Os J Schruers KRJ. The association between social phobia, social anxiety cognitions and paranoid symptoms. Acta Psychiatrica Scandinavica. (2011) 125:213–27. 10.1111/j.1600-0447.2011.01787.x 22077136

[15] Wittchen HU Fuetsch M Sonntag H Müller N Liebowitz M. Disability and quality of life in pure and comorbid social phobia. Findings from a controlled study. Eur Psychiatry. (2000) 15:46–58. 10.1016/S0924-9338(00)00211-X 10713802

[16] Woodward LJ Fergusson DM. Life course outcomes of young people with anxiety disorders in adolescence. J Am Acad Child Adolesc Psychiatry. (2001) 40:1086–93. 10.1097/00004583-200109000-00018 11556633

[17] Buchanan T Smith JL. Using the Internet for psychological research: personality testing on the World Wide Web. Br J Psychol. (1999) 90:125–44. 10.1348/000712699161189 10085550

[18] Carlbring P Brunt S Bohman S Austin D Richards J Öst LG. Internet vs. paper and pencil administration of questionnaires commonly used in panic/agoraphobia research. Comput Hum Behav. (2007) 23:1421–34. 10.1016/j.chb.2005.05.002

[19] Li S Wang Y Xue J Zhao N Zhu T. The impact of COVID-19 epidemic declaration on psychological consequences: a study on active Weibo users. Int J Environ Res Public Health. (2020) 17:2032. 10.3390/ijerph17062032 32204411 PMC7143846

[20] Weibo Data Center. 2020 Weibo User Development Report. (2020). Available online at: http://k.sina.com.cn/article_6519757211_1849b999b02001ju6q.html (accessed July 3, 2022).

[21] Golder SA Macy MW. Diurnal and seasonal mood vary with work, sleep, and daylength across diverse cultures. Science. (2011) 333:1878–81. 10.1126/science.1202775 21960633

[22] Salathé M Khandelwal S. Assessing vaccination sentiments with online social media: implications for infectious disease dynamics and control. PLoS Comput Biol. (2011) 7:e1002199. 10.1371/journal.pcbi.1002199 22022249 PMC3192813

[23] Signorini A Segre AM Polgreen PM. The use of twitter to track levels of disease activity and public concern in the us During the influenza a H1N1 pandemic. PLoS ONE. (2011) 6:e19467. 10.1371/journal.pone.0019467 21573238 PMC3087759

[24] Qiu L Lin H Ramsay J Yang F. You are what you tweet: personality expression and perception on Twitter. J Res Pers. (2012) 46:710–8. 10.1016/j.jrp.2012.08.008

[25] Li A Hao B Bai S Zhu T. Predicting psychological features based on web behavioral data: Mental health status and subjective well-being. Chin Sci Bullet. (2015) 60:994–1001. 10.1360/N972014-00763

[26] Erwin BA Turk CL Heimberg RG Fresco DM Hantula DA. The Internet: home to severe population of individuals with social anxiety disorder? Anxiety Disord. (2004) 18:629–46. 10.1016/j.janxdis.2003.08.002 15275943

[27] Caplan SE. Relations among loneliness, social anxiety, and problematic Internet use. Cyberpsychol Behav. (2007) 10:234–42. 10.1089/cpb.2006.9963 17474841

[28] Jia Y Jia Y. Neuroticism and mobile social network usage preference of college students: the mediating role of social anxiety. Chin J Clin Psychol. (2020) 28:285–8.

[29] Caplan SE. A social skill account of problematic Internet use. J Commun. (2005) 55:721–36. 10.1111/j.1460-2466.2005.tb03019.x

[30] McKenna KYA Green AS Gleason MEJ. Relationship formation on the internet: what's the big attraction? J Soc Iss. (2002) 58:9–31. 10.1111/1540-4560.00246

[31] Wang X Wang Y Zhou M Li B Liu X Zhu T. Identifying psychological symptoms based on facial movements. Front Psychiatry. (2020) 11:607890. 10.3389/fpsyt.2020.607890 33384632 PMC7769937

[32] Vallejo MA Jordán CM Díaz MI Comeche MI Ortega J. Psychological assessment via the internet: a reliability and validity study of online (vs paper-and-pencil) versions of the General Health Questionnaire-28 (GHQ-28) and the Symptoms Check-List-90-Revised (SCL-90-R). J Med Internet Res. (2007) 9:e2. 10.2196/jmir.9.1.e2 17478411 PMC1794673

[33] Zheng Y Zhao J Phillips M Liu J Cai M Sun S. Validity and reliability of the Chinese Hamilton Depression Rating Scale. Br J Psychiatry. (1988) 152:660–4. 10.1192/bjp.152.5.660 3167442

[34] Leary MR Kowalski RM. The Interaction Anxiousness Scale: construct and criterion-related validity. J Pers Assess. (1993) 61:136–46. 10.1207/s15327752jpa6101_10 8377098

[35] Gao R Hao BB Li H Gao YS Zhu TS. Developing simplified Chinese psychological linguistic analysis dictionary for microblog. In: International Conference on Brain and Health Informatics. Berlin: Springer-Cham. (2013). p. 359–368. 10.1007/978-3-319-02753-1_36

[36] Liu T Che W Li Z. The linguistic platform technology. J Chin Inform Proces. (2011) 6:53–62.

[37] Liu M Xue J Zhao N Wang X Jiao D Zhu T. Using social media to explore the consequences of domestic violence on mental health. J Interpers Violence. (2018) 2:1–21. 10.1177/0886260518757756 29441804

[38] Zhao N Jiao D Bai S Zhu T. Evaluating the validity of simplified chinese version of LIWC in detecting psychological expressions in short texts on social network services. PLoS ONE. (2016) 11:e0157947. 10.1371/journal.pone.0157947 27322382 PMC4920595

[39] Zhang Y Yu F. Which socio-economic indicators influence collective morality? Big data analysis on online Chinese social media. Emerg Market Fin Trade. (2018) 54:792–800. 10.1080/1540496X.2017.1321984

[40] Wu S Yang C Zhang Y. The Chinese version of moral foundations dictionary: a brief introduction and pilot analysis. ChinaXiv. (2019). 10.12074/201911.00002

[41] Ren X Xiang Y Zhou Y Zhu T. Individualism/collectivism map of China based on Weibo. J Inner Mongolia Normal Univ. (2017) 46:59–64.

[42] Lv M Li A Liu T Zhu T. Creating a Chinese suicide dictionary for identifying suicide risk on social media. PeerJ. (2015) 3:e1455. 10.7717/peerj.1455 26713232 PMC4690390

[43] Dong Y Chen H Lai K Yue G. Weibo social moods measurement and validation. J Psycholog Sci. (2015) 38:1141–6. 10.16719/j.cnki.1671-6981.2015.05.034

[44] Fincher CL Thornhill R. Parasite-stress promotes in-group assortative sociality: the cases of strong family ties and heightened religiosity. Behav Brain Sci. (2012) 35:61–79. 10.1017/S0140525X11000021 22289223

[45] Asada K Tojo Y Osanai H Saito A Hasegawa T Kumagaya S. Reduced personal space in individuals with autism spectrum disorder. PLoS ONE. (2016) 11:e0146306. 10.1371/journal.pone.0146306 26814479 PMC4729526

[46] Hui Q He A Li Q. Cross-lagged regression analysis of gratitude and interaction anxiousness. Educat Res Exp. (2018) 4:84–7.

[47] Zhu K Lu B Chen M Li P Zhong S Lv F. Research on relationship between family environment and mental health of college students. Chin J Behav Medical Sci. (2003) 12:574–5.

[48] Kashdan TB Herbert JD. Social anxiety disorder in childhood and adolescence: current status and future directions. Clin Child Fam Psychol Rev. (2001) 4:37–61. 10.1023/A:1009576610507 11388563

[49] Kashdan TB Steger MF. Expanding the topography of social anxiety. An experience-sampling assessment of positive emotions, positive events, and emotion suppression. Psychol Sci. (2006) 17:120–8. 10.1111/j.1467-9280.2006.01674.x 16466419

[50] Nezlek JB Kuppens P. Regulating positive and negative emotions in daily life. J Pers. (2008) 76:561–80. 10.1111/j.1467-6494.2008.00496.x 18399953

[51] Guo Y Li X Zhang Z Cui H. Personality traits and emotion regulation in patients with anxiety disorder. In: Paper Presented at the 15th National Aged Care Academic Exchange Conference, Liaoning, China (2012).

[52] Bailey ER Matz SC Youyou W Iyengar SS. Authentic self-expression on social media is associated with greater subjective well-being. Nat Commun. (2020) 11:4889. 10.1038/s41467-020-18539-w 33024115 PMC7538578

[53] Graham JR. Assessing personality and psychopathology with interviews. In: Weiner IB Graham JR Naglieri JA, editors, Handbook of Psychology (Vol10). Hoboken: John Wiley and Sons, Inc. (2003). p. 1.

[54] Li L Li A Hao B Guan Z Zhu T. Predicting active users' personality based on micro-blogging behaviors. PLoS ONE. (2014) 9:e84997. 10.1371/journal.pone.0084997 24465462 PMC3898945

[55] Su Y Liu M Zhao N Liu X Zhu T. Identifying psychological indexes based on social media data: a machine learning method. Adv Psycholog Sci. (2021) 29:571–85. 10.3724/SP.J.1042.2021.00571

[56] Zhu T Wang J Zhao N Liu X. Reform on psychological research in big data age. J Xinjiang Normal Univ. (2015) 36:100–7.

[57] Liu Z Geng H Chen H Zhu M Zhu T. Exploring the mechanisms of influence on COVID-19 preventive behaviors in China's social media users. Int J Environ Res Public Health. (2020) 17:8766. 10.3390/ijerph17238766 33255768 PMC7728355

